# Mechanical testing dataset of cast copper alloys for the purpose of digitalization

**DOI:** 10.1016/j.dib.2024.110687

**Published:** 2024-06-26

**Authors:** Hossein Beygi Nasrabadi, Felix Bauer, Patrick Uhlemann, Steffen Thärig, Birgit Rehmer, Birgit Skrotzki

**Affiliations:** a Bundesanstalt für Materialforschung und -prüfung (BAM), Berlin, Germany; b fem Research Institute (fem), Schwäbisch Gmünd, Germany

**Keywords:** FAIR principles, Hardness, Tensile testing, Stress relaxation, Low-Cycle Fatigue (LCF)

## Abstract

This data article presents a set of primary, analyzed, and digitalized mechanical testing datasets for nine copper alloys. The mechanical testing methods including the Brinell and Vickers hardness, tensile, stress relaxation, and low-cycle fatigue (LCF) testing were performed according to the DIN/ISO standards. The obtained primary testing data (84 files) mainly contain the raw measured data along with the testing metadata of the processes, materials, and testing machines. Five secondary datasets were also provided for each testing method by collecting the main meta- and measurement data from the primary data and the outputs of data analyses. These datasets give materials scientists beneficial data for comparative material selection analyses by clarifying the wide range of mechanical properties of copper alloys, including Brinell and Vickers hardness, yield and tensile strengths, elongation, reduction of area, relaxed and residual stresses, and LCF fatigue life. Furthermore, both the primary and secondary datasets were digitalized by the approach introduced in the research article entitled “Toward a digital materials mechanical testing lab” [1]. The resulting open-linked data are the machine-processable semantic descriptions of data and their generation processes and can be easily queried by semantic searches to enable advanced data-driven materials research.

Specifications Table

**Table:** 

Subject	Materials Science/ Material Characterization
Specific subject area	Different mechanical testing methods for characterizing the mechanical properties of several copper alloys and digitalization of the test data
Type of data	Table, Figure, Dataset, Turtle files Primary, Analyzed, Digitalized
Data collection	Brinell hardness testing according to the DIN EN ISO 6506-1:2015 standard [2] and using an Emco Test M4C 025 G3 machine. Vickers hardness testing according to the DIN EN ISO 6507-1:2018 standard [3] and using a KB 30 SR FA Basic machine. Tensile testing according to the DIN EN ISO 6892-1:2020 standard [4] and stress relaxation tests according to the DIN EN 10319-1:2003 standard [5], both by MTS Test System Model C45.105 tensile testing machine. LCF tests according to ISO 12106:2017-03 standard [6] and by Instron 8561 testing machine. Digitalization of the test data by the approach introduced in [1].
Data source location	Bundesanstalt für Materialforschung und -prüfung (BAM), Division 5.2, 12205 Berlin, Germany
Data accessibility	Repository name: Zenodo, CKAN Data identification number: 10.5281/zenodo.7670582 and 10.5281/zenodo.10820437 Direct URL to data: https://zenodo.org/records/10820299 and https://zenodo.org/records/10820438 All the primary, secondary, and digitalized datasets are also publicly available at CKAN: https://ckan.kupferdigital.org/dataset/?organization=bam
Related research article	H. Beygi Nasrabadi, T. Hanke, M. Weber, M. Eisenbart, F. Bauer, R. Meissner, G. Dziwis, L. Tikana, Y. Chen, B. Skrotzki, Toward a digital materials mechanical testing lab, Computers in Industry 153 (2023) 104016 https://doi.org/10.1016/j.compind.2023.104016

## 1 Value of the Data

• The datasets presented in this article collected the primary and analyzed data from different mechanical testing methods (Brinell and Vickers hardness, tensile, stress relaxation, and low-cycle fatigue) of several copper alloys. All the testing procedures were performed according to the DIN/ISO test standards, and the test data involved the most possible metadata about the process, material, machines, and equipment calibration. As most of these testing methods require special testing devices and expertise or time-consuming measurements, the presented datasets are of interest to both materials science and industry.
• The dataset provides an insight into the wide variety of mechanical properties of copper alloys (hardness, yield strength, tensile strength, modulus, reduction of area, elongation, relaxed/residual stress, and LCF fatigue life) that help materials scientists and the copper industry for comparative material selection analyses and development of more functional copper parts like bearings and gears.
• The datasets are beneficial to researchers wishing to develop or verify materials deformation models, organize datasets for numerical component assessment, or cross-reference with measurements from other techniques for equipment/methodological assessment.
• The open-linked data which are the machine-processable semantic descriptions of data and their generation processes can be easily queried using advanced semantic searches and enable machines to prepare different types of datasets for data-driven research like machine learning [7].
• The digital dataset provides an example of the digitalization of data-driven industries and the further growth of Industry 4.0 technologies [8]. This approach gives the industry access to a substantial amount of trustworthy and traceable mechanical testing data of other academic and industrial institutions and organizes various data-driven research for increasing productivity and production efficiency, reducing manufacturing costs, and designing new functional products.

## 2 Background

Mechanical testing datasets are generated daily in thousands of materials testing labs around the world. However, most of these datasets do not meet the criteria of being Findable, Accessible, Interoperable, and Reusable (FAIR), and thus cannot be (re-)used for data-driven product development [9]. Digitalization of the mechanical testing data and their storage by a standardized structure in publicly available repositories has been introduced as an advantageous method for the generation of highly FAIR testing datasets [10]. Here, the main requirements for such a digitalization process are: i) providing the test data according to the testing standards and along with all required process metadata, ii) developing the mechanical testing knowledge graphs and ontologies, and iii) utilizing suitable data management approaches for mapping the test data to knowledge graphs, their conversion to the Resource Description Framework (RDF) data model, and storage in public and queryable repositories [1]. In a recently published paper [1], we provided all the above requirements for the digitalization of example testing data. The current repository gathered several datasets from different mechanical testing methods of various copper alloys, which enable materials scientists to perform advanced semantic queries and further material developments.

## 3 Data Description

**Fig. 1:**
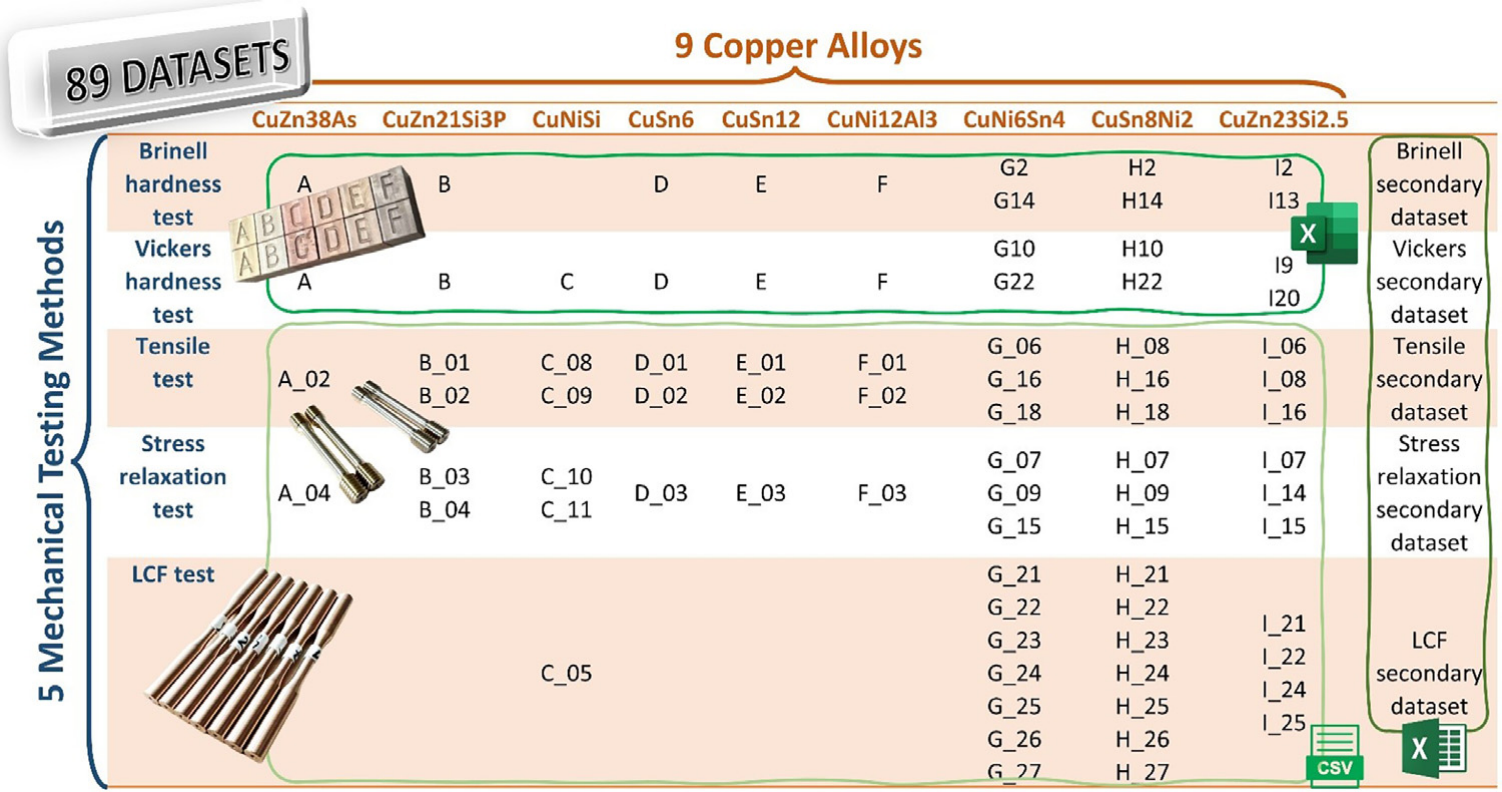
Overview of the datasets for mechanical testing of the examined copper alloys.

This section describes the mechanical testing data of several copper alloys which were provided in two repositories [11,12] (see Data accessibility in the Specification Table above). Overall, five folders represent five distinct mechanical testing methods: Brinell hardness, Vickers hardness, and tensile tests in the first repository; and stress relaxation and LCF tests in the second one. Fig. 1 lists all the files that have been preserved in the aforementioned repositories. As can be seen, each folder comprised two sub-folders of primary and secondary data. The primary data are the raw data (*.xlsx or *.lis files) given by the testing laboratories for each sample, whereas the secondary data are tabular *.xlsx sheets that aggregate the main metadata and the analyzed data of all samples.

**Brinell and Vickers hardness data:** In the case of the Brinell hardness test, the primary data folder contains 11 *.xlsx files which were named by their sample IDs (e.g., A, B, D, etc.). The data reported in each primary file can be categorized into six sections: provenance metadata (such as used test standard and date); measurement metadata (such as load, indenter diameter, and calibration values); measured primary data (such as the vertical and horizontal diameters of various indentations); measurement secondary data (such as the average diameter of indentations); and analyzed secondary data (such as final Brinell hardness and uncertainty of the sample).

The primary Vickers hardness data consists of 12 *.xlsx files with the same format as the Brinell test data. The differences between the two testing reports are related to the shape of the indenters, the applied loads and times, the diameters of the circular Brinell indentations or the diagonal of the pyramidal-like Vickers indentations, and the test standards and equations for calculating the hardness and uncertainty values. Both the Brinell and Vickers hardness secondary datasets collect the main meta- and measurement data from the primary test reports.

The columns of such datasets contain provenance metadata (test standard and test date), test piece metadata (identifier, composition, provider, preparation, and dimension), testing machine metadata (identifier, indenter material and diameter), certified reference material (CRM) metadata (identifier, certified Brinell hardness, measured average and standard deviation of Brinell hardness, and its uncertainty value and constants), and testing metadata (temperature, applied force, time, and indentations distances). Five to six indentations were created on each sample and their horizontal, vertical, and average diameters, and calculated Brinell hardness values were collected on the secondary dataset. The Brinell hardness uncertainty was determined by calculating the uncertainty values from CRM, testing machine, measurement resolution, as well as the permissible uncertainty. Eventually, the final hardness values were measured by summation of both the Brinell hardness averages and their uncertainties.

**Fig. 2:**
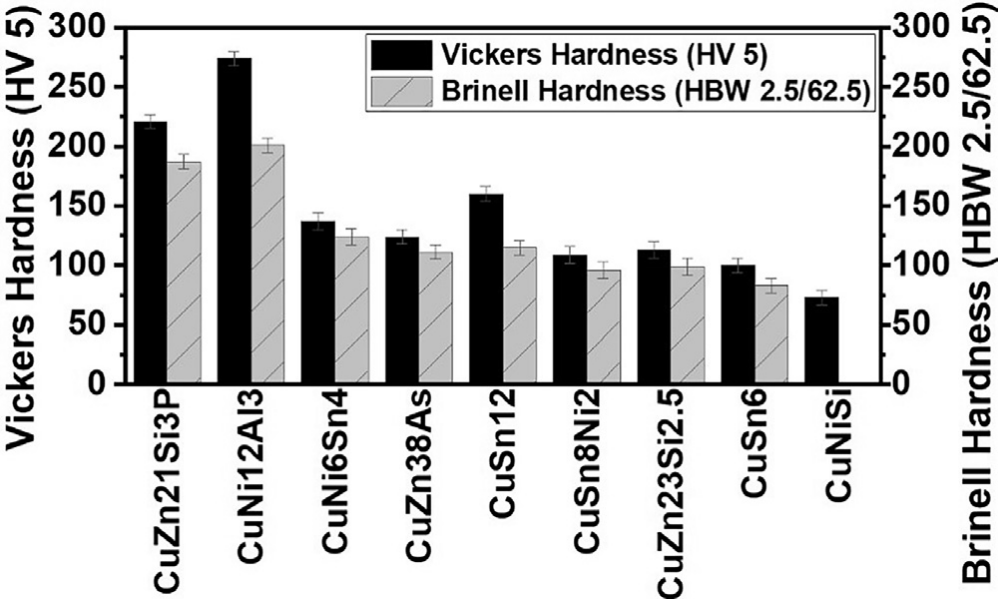
Brinell and Vickers hardness values of different copper alloys.

The structure of the Vickers secondary dataset and calculation of the final hardness values follow the one of the Brinell secondary dataset. A small part of such comprehensive secondary Brinell and Vickers datasets is shown in Table 1. As an important scientific output of these hardness testing methods, Fig. 2 plots the variation of Brinell and Vickers harnesses for different copper alloys. This sort of plot allows material scientists to better understand how alloy composition and alloying elements influence copper alloy hardness variation.

**Table 1:** Dataset for the Brinell and Vickers hardness testing of copper alloys.

Sample	Average Brinell hardness (HBW 2.5/62.5)	Standard deviation Brinell hardness (HBW 2.5/62.5)	Brinell hardness uncertainty (HBW 2.5/62.5)	Final Brinell hardness and uncertainty (HBW 2.5/62.5)	Average Vickers hardness (HV 5)	Standard deviation Vickers hardness (HV 5)	Vickers hardness uncertainty (HV 5)	Final Vickers hardness and uncertainty (HV 5)
CuZn38As	111.48	6.71	5.5	111 ± 6	124.3	1.6	5.9	124 ± 6
CuZn21Si3P	186.45	9.34	5.5	187 ± 6	221.5	2.1	5.9	221± 6
CuNiSi	–	–	–	–	72.7	2.5	5.9	73 ± 6
CuSn6	82.60	10.22	5.5	83 ± 6	100.3	7.9	5.9	100 ± 6
CuSn12	115.28	8.81	5.5	115 ± 6	159.7	4.6	5.9	160 ± 6
CuNi12Al3	201.23	4.69	5.5	201 ± 6	273.6	11.1	5.9	274 ± 6
CuNi6Sn4	124.12	10.23	6.5	124± 7	115.1	7.9	6.6	115 ± 7
CuNi6Sn4	123.91	10.09	6.5	124± 7	137.2	7.9	6.6	137 ± 7
CuSn8Ni2	97.64	5.44	6.5	98± 7	110.3	3.0	6.6	110 ± 7
CuSn8Ni2	94.23	4.72	6.5	94± 7	108.7	3.4	6.6	109 ± 7
CuZn23Si2.5	141.01	3.42	6.5	141± 7*	115.5	1.1	6.6	116 ± 7
CuZn23Si2.5	99.26	4.19	6.5	99± 7*	109.4	2.3	6.6	109 ± 7

⁎ The Brinell hardness variation in CuZn23Si2.5 samples can be explained by the Zinc evaporation during the casting process resulting in chemical composition changes along the continuous cast bar.

**Fig. 3:**
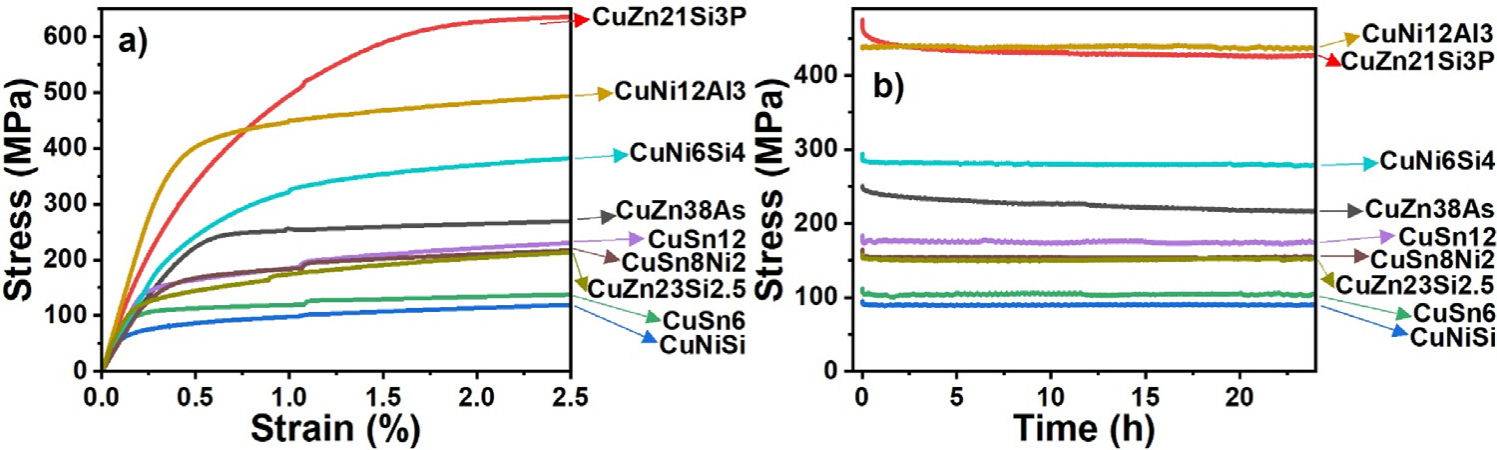
a) Exemplary stress-strain curves of different copper alloys and b) stress relaxation over time for different copper alloys (at 100 °C).

**Tensile and stress relaxation data:** The tensile test/primary data folder contains 20 primary data from tensile testing of different copper alloys. All the primary data have *.lis file format, a tab-delimited format that can be opened like ASCII ∗.txt or ∗.dat files by e.g., text editors, Excel, or common data analysis software. Each primary data file consists of two parts: measurement metadata (experiment name, test date, project name, material, operator, testing machine, sample ID, sample cross-section, test temperature, test speed, initial measurement length, measured slope of the elastic part (m_E_), yield strength (R_p0.2_), tensile strength, elongation at fracture, and reduction of area) and raw test data (time, displacement, load, strain, and stress values). The raw data can be used for plotting the stress-strain curves of different copper alloys (Fig. 3a). Most of the important mechanical properties of materials (like the slope of the elastic region and yield strength) can be obtained by analyzing these stress-strain curves. The tensile secondary dataset.xlsx has been prepared by collecting the main measurement metadata and the abovementioned analyzed data. Table 2 represents a part of such a secondary dataset for the tensile testing technique, including the values of yield strength, slope of elastic region, ultimate tensile strength, elongation after fracture, and reduction of area of the tested copper samples.

**Table 2:** Dataset for the mechanical properties of different copper samples obtained from tensile testing.

Sample	Yield strength R_p0.2_ (MPa)	Slope of elastic region m_E_ (GPa)	Ultimate tensile strength (MPa)	Elongation after fracture (%)	Reduction of area (%)
A_02	240	59.5	348	36.5	69
B_01	334	104	680	24.5	38
B_02	352	106	682	25.5	37
C_08	80.7	64.9	236	43.0	88
C_09	79.1	68.5	207	39.0	83
D_01	119	58.8	314	33.5	77
D_02	111	71.0	261	43.5	74
E_01	169	102	331	11.0	10
E_02	160	70.4	380	39.0	39
F_01	409	114	637	24.5	37
F_02	413	114	639	19.0	28
G_06	242	66.3	444	26.0	75
G_16	269	63.5	480	22.0	76
G_18	300	65.6	525	27.5	72
H_08	167	56.2	386	41.0	69
H_16	167	58.2	385	39.0	72
H_18	168	57.9	393	40.0	70
I_06	162	121	406	13.0	17
I_08	137	86.5	422	33.5	33
I_16	134	90.7	423	46.0	48

Both the primary and secondary data from the stress relaxation tests have a similar structure as the tensile test data. The stress relaxation test is a kind of tensile test that is stopped after reaching ≈ 1 % strain, and then the sample is held for 24 h at this strain and constant temperature. Therefore, like primary tensile test data, the primary stress relaxation test data have two parts of measurement metadata and raw test data. It should be mentioned that the raw test data of the tensile stress relaxations test were partially edited by the machine operator: the stress-strain curves were shifted such that the fit to the elastic range of the curve passes to the zero point. In addition, the measured data after 24 h of relaxation were deleted. Fig. 3b is a plot of the stress decay over the relaxation time. This plot can provide information about the trend of stress relaxation with time and the amount of relaxed/residual stress within each copper alloy after the standardized test process. Note that the measured force and calculated stress data are somehow noisy due to very small variations in the extensometer cooling water temperature (up to ±1 °C).

The stress relaxation secondary dataset was prepared by collecting the measurement metadata and the analyzed metadata for 17 tests performed on different copper alloys. The column of such dataset contains provenance metadata (test standard and test date), test piece metadata (identifier, composition, provider, preparation, cross-section shape, total length, parallel length, cross-section area), testing machine metadata (identifier), testing process metadata (test temperature, time, strain rate, initial strain/stress), and analyzed metadata (yield strength, slope of elastic region, relaxed/residual stresses). Note that the values given for the slope of the elastic region, m_E_, and yield strength, R_p0.2_, are for information only, as their evaluation is not intended according to the DIN EN 10319-1:2003 standard. Part of the stress relaxation secondary dataset is presented in Table 3. Deviating from DIN EN 10319-1:2003, in some cases the residual stress given in Table 3 is not the remaining stress after 24 h, but the minimum stress during the test. However, the differences are very small.

**Table 3:** Dataset for the tensile stress relaxation testing of copper samples.

Sample	Initial Strain (%)	Initial Stress (MPa)	Residual Stress after 24 h at 100 °C (MPa)	Relaxed Stress after 24 h at 100 °C (%)
A_04	1.00	250	216	13.6
B_03	1.00	474	419	11.6
B_04	1.00	475	424	10.7
C_10	1.00	95.1	87.9	7.6
C_11	0.99	98.6	91.4	7.3
D_03	1.01	113	100	11.5
E_03	0.99	184	171	7.1
F_03	0.99	441	435	1.4
G_07	0.99	292	277	5.1
G_09	1.01	308	291	5.5
G_15	1.01	249	235	5.6
H_07	1.02	164	152	7.3
H_09	1.01	161	149	7.5
H_15	1.01	154	144	6.5
I_07	1.01	167	161	3.6
I_14	1.00	158	149	5.7
I_15	1.01	157	145	7.6

**Fig. 4:**
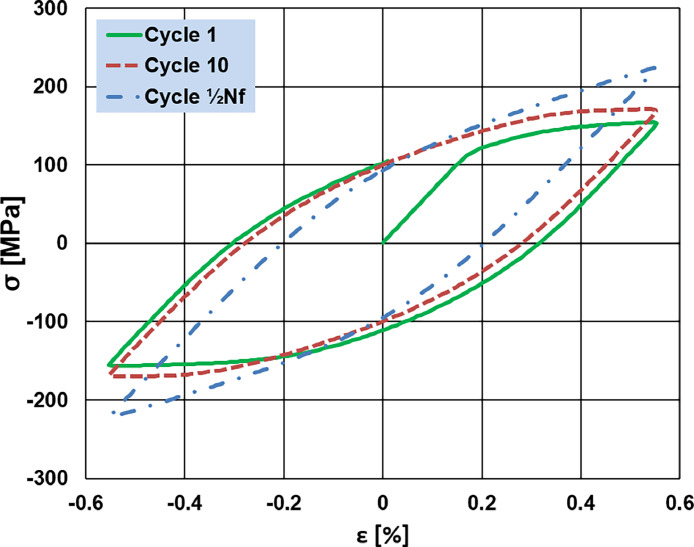
Stress-strain hysteresis for the 1st and 10th cycle and the cycle at half-lifetime for sample H_21.

**LCF data:** The folder LCF test/primary data contained 19 primary LCF data acquired from LCF testing of four copper alloys (IDs of C, G, H, and I) at various strain ranges (e_max_ - e_min_). All the primary data have *.lis format and include two types of data: testing metadata (provenance, test price, testing machine, and testing parameters metadata as well as the number of cycles to failure (N_f10__%_), the total number of fatigue cycles (N) and fatigue location) and raw measurement data (maximum and minimum values of stress, and strain, for each cycle). The LCF secondary dataset has been prepared by collecting the main measurement metadata and analyzing data from all experiments. Part of the LCF secondary dataset is shown in Table 4. The full dataset contains the following columns: test standard, test date, test piece metadata (identifier, composition, provider, preparation, cross-section shape, total length, parallel length, original cross-section area), testing machine identifier, ambient medium, test process metadata (temperature, strain rate, maximum/minimum strain, strain ratio, cycle time), fatigue life, cycles at the end of the experiment, and fracture location. It should be noted that all the LCF tests were performed in the strain-controlled mode, meaning that the max/min strain was kept constant during each test process and the stress variations were recorded for each cycle (Fig. 4). Eventually, the N_f_ value was determined using the failure criterium of 10 % load drop of the maximum peak stress in the cyclic stress response curve vs. the number of cycles [6].

**Table 4:** Dataset for fatigue testing criteria and corresponding failure cycles of copper alloys.

Sample	Strain rate (%/s)	e_max_	e_min_	Strain ratio Re	Cycle time (s)	Fatigue life, N_f10__%_ (-)
C-05	0.1	0.40	−0.40	−1	16	47,758
G_21	0.1	0.50	−0.50	−1	20	27,813
G_22	0.1	0.45	−0.45	−1	18	8183
G_23	0.1	0.40	−0.40	−1	16	7868
G_24	0.1	0.35	−0.35	−1	14	12,422
G_25	0.1	0.50	−0.50	−1	20	3291
G_26	0.1	0.45	−0.45	−1	18	5617
G_27	0.1	0.40	−0.40	−1	16	8788
H_21	0.1	0.55	−0.55	−1	22	21,040
H_22	0.1	0.45	−0.45	−1	18	35,640
H_23	0.1	0.60	−0.60	−1	24	13,463
H_24	0.1	0.35	−0.35	−1	14	run out*
H_25	0.1	0.50	−0.50	−1	20	29,038
H_26	0.1	0.45	−0.45	−1	18	65,372
H_27	0.1	0.40	−0.40	−1	16	run out*
I_21	0.1	0.50	−0.50	−1	20	9726
I_22	0.1	0.45	−0.45	−1	18	15,576
I_24	0.1	0.35	−0.35	−1	14	50,749
I_25	0.1	0.40	−0.40	−1	16	20,789

⁎ More than 100,000 cycles were defined as run out.

**Fig. 5:**
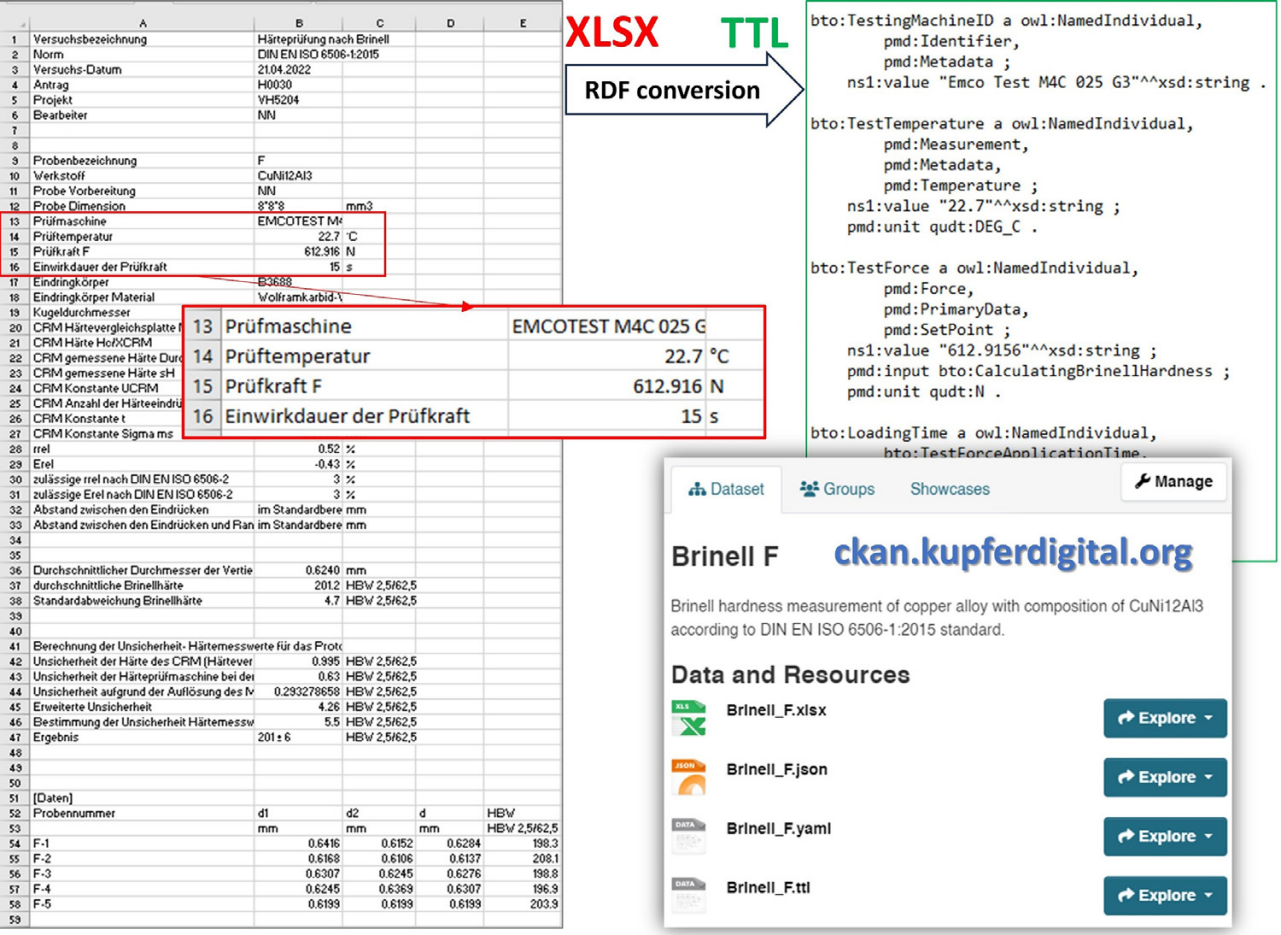
Conversion of example Brinell test primary data from *.xlsx to *.ttl schema and management of digital datasets in CKAN.

**Digitalized data:** All 89 datasets of Fig. 1 were converted to the digital *.ttl format using the CKAN approach [1]. TTL (turtle) stands for “Terse RDF Triple Language” and is a W3C standard that describes itself as a general-purpose language for describing information on the web [13]. Fig. 5 shows an example of conversion of *.xlsx-type Brinell test primary data to *.ttl schema. The *.ttl files were developed in such a way to represent all the data linked to their testing processes, so any relations between various kinds of data and testing variables were also included in the converted files. Here, all the entities of *.ttl test data were described by the Web Ontology Language (OWL) [14] and utilized the vocabularies of PROV-O^1^ (PROV Ontology), PMDco^2^ (Platform Material Digital core ontology), and QUDT^3^ (Quantities, Units, Dimensions, and data Types ontologies). Such linked data are completely machine-readable and along with the source *.xlsx or *.lis files were published in the CKAN^4^ (see the bottom right part of Fig. 5). The digitalized and open linked mechanical testing data in publicly available CKAN data space have high FAIR score and can be easily queried by the semantic query languages (SQLs) [15], and reused for data-driven materials research, prediction, or product development purposes.

## 4 Experimental Design, Materials and Methods

### 4.1 Materials

Table 5 represents the composition and processing method of different cast copper alloys supplied by the fem Research Institute (fem, Germany) and the German Copper Institute (DKI, Germany).

**Table 5:** Description of the nominal composition, provider, and processing method of different copper alloys that were used for the preparation of the mechanical testing datasets.

ID	Alloy composition	Provider	Processing method
A	CuZn38As	DKI	Casting, hot and cold extrusion
B	CuZn21Si3P	DKI	Casting and hot extrusion
C	CuNiSi	fem	Continuous casting
D	CuSn6	fem	Continuous casting
E	CuSn12	fem	Continuous casting
F	CuNi12Al3	fem	Continuous casting
G	CuNi6Sn4	fem	Continuous casting + heat treatment*
H	CuSn8Ni2	fem	Continuous casting
I	CuZn23Si2.5	fem	Continuous casting

⁎ The heat treatment consisted of a heating step at 800 °C for 40 min followed by the water quenching, and another heating step at 410 °C for 3 h.

### 4.2 Mechanical testing

All the mechanical testing experiments in this section were carried out according to DIN/ISO testing standards in the accredited materials testing laboratory of the “Bundesanstalt für Materialforschung und -prüfung (BAM)”.

**Brinell hardness testing:** Brinell hardness tests were performed according to the DIN EN ISO 6506-1:2015 standard [2]. For these experiments, the samples of cubic (8 × 8 × 8 mm^3^) or cylinder shape (diameter and height of 18 mm and 15 mm, respectively) were cut, smoothed, polished, and cleaned according to the method mentioned in the test standard. The Brinell hardness tests were done using an Emco Test M4C 025 G3 machine, equipped with a spherical tungsten carbide composite indenter (ID: B3688, diameter: 2.5 mm). The tests were performed at room temperature by applying a test force of 612.9156N and a loading time of 14 s. 5–6 indentations were created on each sample, while the distances between the indentations (and between the sample edges and indentations) met the requirements of the test standard. Note that the hardness value of sample C was not reported due to a measurement error. Furthermore, for validation of the testing machine and determining the test uncertainties, the Brinell hardness of a Certified Reference Material (CRM, ID:15.808.010.607) was measured five times by testing with similar parameters. The CRM has a certified Brinell hardness of 142 HBW 2.5/62.5, and its U_CRM_ (measurement uncertainty of the hardness reference block), t (uncertainty of CRM hardness measurement replications), δ_ms_ (resolution of the hardness testing machine), and E_rel_ (permissible error) constants are respectively 1.99, 1.14, 0.00155, and 3.

**Vickers hardness testing:** Vickers hardness tests were carried out in compliance with DIN EN ISO 6507-1:2018 [3]. For these experiments, the samples with similar dimensions to the Brinell hardness test were prepared according to the test standard requirements. The Vickers hardness tests were done using a KB 30 SR FA Basic machine, equipped with a diamond indenter (angle: 136°). The tests were performed at room temperature, applying a test force of 49.03N and a loading time of 14 s. 5–6 indentations were created on each sample, while the distances between the indentations (and between the sample edges and indentations) met the requirements of the test standard. Furthermore, for validation of the testing machine and measuring the test uncertainties, a CRM (ID: 8890101.0620) Vickers hardness was measured by testing with similar parameters. This piece's certified Vickers hardness, U_CRM_, and δ_ms_ constants are 213 HV 5, 2.4 HV 5, and 0.00026, respectively.

**Fig. 6:**
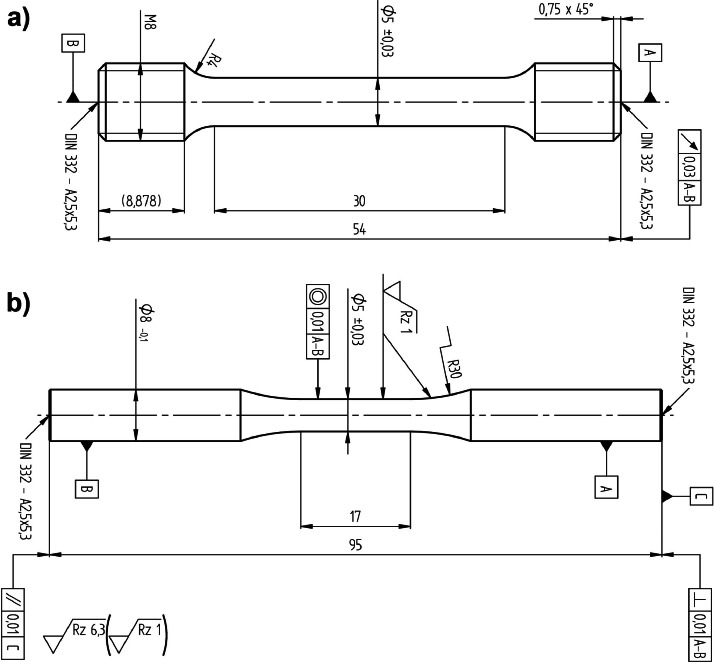
Dimensions of the machined tensile (a) and LCF (b) test specimens.

**Tensile testing:** Tensile test experiments were done according to the DIN EN ISO 6892-1:2020 standard [4]. The shape and dimensions of the test specimens that were prepared for these experiments are shown in Fig. 6a. As can be seen in this image, the test pieces have a cylindrical cross-section and a total length of 54 mm (parallel length of 30 mm). The MTS Test System Model C45.105 tensile testing apparatus (class 1 calibration) and the HBM-DD1 displacement transducer (class 0.5 calibration) were used to conduct the tensile tests. All the tensile test measurements were done at room temperature with a strain rate of 0.025 %/s.

**Tensile stress relaxation testing:** The tensile stress relaxation tests were performed according to the DIN EN 10319-1:2003 standard [5]. The test specimens had the same shape and dimensions as the tensile test specimens (cylindrical cross-section and a total length of 54 mm, Fig. 6a). The tensile stress relaxation tests were done in the air on a 100 kN electromechanical testing machine (MTS Systems, Model C45.105; class 1 calibration) with a strain rate of 0.025 %/s. An axial extensometer (MTS Systems; type 632.51C-05; class 0.5 calibration) of 21 mm nominal gauge length was used. Stress relaxation started after reaching 1 % total strain and continued for 24 h while the temperature was kept constant at 100 °C.

**LCF testing:** The ISO 12106:2017 standard was followed for carrying out the LCF tests [6]. Fig. 6b displays the dimensions of the test specimens that were machined for these measurements. LCF tests were done on a 100 kN electromechanical testing machine (Instron; type 8561; class 1 calibration) in the air at room temperature. An axial extensometer (MTS Systems; type 632.51C-04; 12 mm nominal gauge length; class 1 calibration) was used. The tests were performed at different strain amplitudes at a strain ratio (*R_e_* = e_min_/e_max_) of −1 and a strain rate of 10^−3^/s, respectively.

### 4.3 Converting test reports to the digital twin data

The method for converting the *.lis or *.xlsx-typed mechanical testing data to machine-readable RDF data has been reported in our previous paper [1]. Here, the knowledge graphs of different mechanical testing processes were developed by ontology-based representation of testing entities described in the DIN/ISO testing standards. In the next step, the *.lis or *.xlsx testing reports were mapped into their knowledge graphs via an online mapping tool.^5^ Subsequently, the data-mapped knowledge graphs are converted to machine-readable RDF data by the RDF converter tool.^6^ Such RDF test data were finally uploaded to the CKAN-data management repository,^7^ where they may be simply processed by a knowledge base triple store.

## Limitations

Not applicable.

## Ethics Statement

The authors have read and followed the ethical requirements for publication in Data in Brief and confirmed that the current work does not involve human subjects, animal experiments, or any data collected from social media platforms.

## CRediT authorship contribution statement

**Hossein Beygi Nasrabadi:** Conceptualization, Methodology, Validation, Formal analysis, Investigation, Data curation, Writing – original draft. **Felix Bauer:** Resources, Writing – review & editing. **Patrick Uhlemann:** Investigation, Data curation, Validation. **Steffen Thärig:** Investigation, Data curation, Validation. **Birgit Rehmer:** Validation, Writing – review & editing. **Birgit Skrotzki:** Conceptualization, Resources, Writing – review & editing, Supervision.

## Data Availability

KupferDigital mechanical testing datasets: Stress relaxation and low-cycle fatigue (LCF) tests (Original data) (Zenodo) KupferDigital mechanical testing datasets: Brinell hardness, Vickers hardness, and tensile tests (Original data) (Zenodo)

## Footnotes

1 https://w3.org/ns/prov#.

2 https://w3id.org/pmd/co/.

3 https://qudt.org/2.1/schema/qudt/.

4 http://ckan.kupferdigital.org/.

5 http://maptomethod.kupferdigital.org/.

6 http://rdfconverter.kupferdigital.org/.

7 http://ckan.kupferdigital.org/.
